# Nicotine affects mitochondrial structure and function in human airway smooth muscle cells

**DOI:** 10.1152/ajplung.00158.2023

**Published:** 2023-11-07

**Authors:** Niyati A. Borkar, Michael A. Thompson, Colleen M. Bartman, Venkatachalem Sathish, Y. S. Prakash, Christina M. Pabelick

**Affiliations:** ^1^Department of Anesthesiology and Perioperative Medicine, Mayo Clinic, Rochester, Minnesota, United States; ^2^Department of Pharmaceutical Sciences, North Dakota State University, Fargo, North Dakota, United States; ^3^Department of Physiology and Biomedical Engineering, Mayo Clinic, Rochester, Minnesota, United States

**Keywords:** asthma, calcium, fission-fusion, mitochondria, nicotinic cholinergic receptor

## Abstract

Exposure to cigarette smoke and e-cigarettes, with nicotine as the active constituent, contributes to increased health risks associated with asthma. Nicotine exerts its functional activity via nicotinic acetylcholine receptors (nAChRs), and the alpha7 subtype (α7nAChR) has recently been shown to adversely affect airway dynamics. The mechanisms of α7nAChR action in airways, particularly in the context of airway smooth muscle (ASM), a key cell type in asthma, are still under investigation. Mitochondria have garnered increasing interest for their role in regulating airway tone and adaptations to cellular stress. Here mitochondrial dynamics such as fusion versus fission, and mitochondrial Ca^2+^ ([Ca^2+^]_m_), play an important role in mitochondrial homeostasis. There is currently no information on effects and mechanisms by which nicotine regulates mitochondrial structure and function in ASM in the context of asthma. We hypothesized that nicotine disrupts mitochondrial morphology, fission-fusion balance, and [Ca^2+^]_m_ regulation, with altered mitochondrial respiration and bioenergetics in the context of asthmatic ASM. Using human ASM (hASM) cells from nonasthmatics, asthmatics, and smokers, we examined the effects of nicotine on mitochondrial dynamics and [Ca^2+^]_m_. Fluorescence [Ca^2+^]_m_ imaging of hASM cells with rhod-2 showed robust responses to 10 μM nicotine, particularly in asthmatics and smokers. In both asthmatics and smokers, nicotine increased the expression of fission proteins while decreasing fusion proteins. Seahorse analysis showed blunted oxidative phosphorylation parameters in response to nicotine in these groups. α7nAChR siRNA blunted nicotine effects, rescuing [Ca^2+^]_m_, changes in mitochondrial structural proteins, and mitochondrial dysfunction. These data highlight mitochondria as a target of nicotine effects on ASM, where mitochondrial disruption and impaired buffering could permit downstream effects of nicotine in the context of asthma.

**NEW & NOTEWORTHY** Asthma is a major healthcare burden, which is further exacerbated by smoking. Recognizing the smoking risk of asthma, understanding the effects of nicotine on asthmatic airways becomes critical. Surprisingly, the mechanisms of nicotine action, even in normal and especially asthmatic airways, are understudied. Accordingly, the goal of this research is to investigate how nicotine influences asthmatic airways in terms of mitochondrial structure and function, via the a7nAChR.

## Graphical abstract

**Figure:**
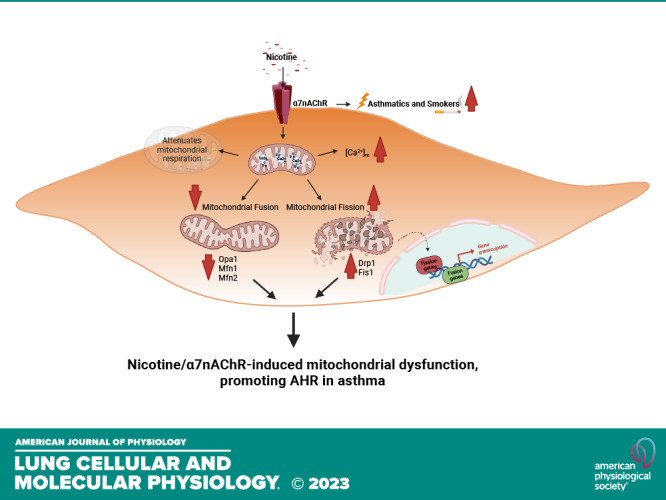

## INTRODUCTION

Airway smooth muscle (ASM) is an important cell type in modulation of airway structure and function (1–5) with effects on airway hyperreactivity, narrowing, and airflow obstruction, as well as to airway remodeling in the context of asthma (6–8). External environmental factors such as tobacco smoke and products of electronic cigarettes (vaping) are known to trigger or exacerbate asthma (9–19). In this regard, even nicotine delivery devices that were previously considered safer for patients with asthma have now been shown not to be so (11, 17–21). Although it is not entirely clear whether it is nicotine per se, the major active element in cigarette smoke and the key ingredient of electronic nicotine delivery devices, nicotine itself has now been shown to enhance airway hyperresponsiveness (12, 14, 19, 22, 23). Importantly, the mechanisms by which nicotine influences airways, including the context of asthma, are still under investigation.

Nicotinic acetylcholine receptors (nAChRs) are known to be present in autonomic ganglia and neuromuscular junctions (24–27). nAChRs are ligand-gated ion channels that regulate neuronal and muscle calcium, neurotransmitter, and cytokine release, and in the long term regulate cell proliferation and other structural aspects (28–30). Relevant to asthma, cholinergic nerves are known to enhance airway contractility and mucus secretion (31–33). Compared with the nervous system, there is relatively little data on nAChRs in nonneuronal cells, including airways. We recently showed that nAChRs are expressed in human ASM, particularly the α7 subunit (α7nAChR), and that via α7nAChR, nicotine can induce cytosolic intracellular Ca^2+^ ([Ca^2+^]_i_) responses (34). Furthermore, we showed that in asthmatic ASM, α7nAChR expression is increased (35), as are nicotine-induced [Ca^2+^]_i_ responses (34). Of the 16 known nAChR subunits (24, 36–38), α7 forms homopentameric nAChRs that exhibit high Ca^2+^ permeability (37) and act as ligand-gated Ca^2+^ channels activated by choline and nicotine, blocked by α-bungarotoxin and methyllycaconitine (MLA; 39–42). In this regard, our previous findings on α7nAChR in human ASM (hASM) make this receptor subtype appealing in the context of airway contractility, consistent with data from cultured rat ASM (43). Whether nAChRs contribute to airway structure or function via other mechanisms remains to be explored.

In addition to their known ligand-gated ion channel function, nAChRs also regulate neurotransmitter and cytokine release, and in the long-term, regulate cell proliferation and other structural aspects (28–30). In this regard, cellular bioenergetics contribute to cell proliferation and other synthetic functions, where mitochondria play a key role. In turn, mitochondrial structure and function (mitochondrial dynamics) are critical factors where a balance between mitochondrial fusion and fission processes helps shape and distribute mitochondria in homeostasis versus adaptation to stress (44–46). Here, proteins such as mitofusin 1 and 2 (Mfn1, Mfn2) and optic atrophy 1 (Opa1) induce fusion of the mitochondrial outer mitochondrial membrane (OMM) and inner mitochondrial membranes (IMM; 47–50). On the other hand, dynamin-related protein 1 (Drp1) is a cytoplasmic protein surrounding the OMM, where it interacts with the fission protein1 (Fis1) to promote mitochondrial fragmentation (49, 51–54). An imbalance between mitochondrial fission, fusion, and bioenergetics is implicated in various diseases (49, 55–59). Here, mitochondria can shift from being sources of energy (e.g., toward contraction or synthetic function) toward triggering cell death in response to external stresses (60). In the context of contraction or synthesis, cytosolic Ca^2+^ is a key determinant, particularly in ASM (7, 61). Studies in different cell types, including ASM, have demonstrated that elevated cytosolic Ca^2+^ leads to increased mitochondrial Ca^2+^ ([Ca^2+^]_m_) (55, 62–66). Furthermore, [Ca^2+^]_m_ can influence cellular respiration, whereas conversely, changes in mitochondrial networks could influence the ability of mitochondria to buffer cytosolic Ca^2+^. Overall, given the interplay between mitochondrial structure and function, in the context of previous findings that nicotine increases [Ca^2+^]_i_, whether nicotine effects further involve [Ca^2+^]_m_ or other mitochondrial parameters is not known. In the present study, we hypothesized that nicotine disrupts mitochondrial fission-fusion balance and influences [Ca^2+^]_m_, altering mitochondrial respiration and bioenergetics. With previous findings of increased nAChRs in asthmatic hASM and in response to cigarette smoke (35), we further explored the idea that in asthmatics or in smokers, such nAChR-mediated effects on mitochondria are enhanced.

## METHODS

### Human ASM

All protocols involving human lung tissue and primary human ASM (hASM) cells were approved by the Mayo Clinic Institutional Review Board (67). Surgical lung specimens of patients undergoing lobectomy for focal, noninfectious disease were obtained following patient written informed consent, and normal areas of third- to sixth-generation bronchi were identified and dissected for further use. Medical records were used to identify asthma status and smoking history. hASM cells were isolated and cultured using previously established procedures with experiments limited to less than five passages of subculture, and cells being serum deprived for at least 24 h, along with consistent verification of smooth muscle phenotype. For both asthmatics and nonasthmatics, samples in either group were used for a range of experimental protocols, although not all patient samples were used for every protocol.

### Cell Treatments

Following serum starvation, cells were treated with nicotine (10 µM). This nicotine concentration was selected based on pilot four-log dose-response studies showing maximal responses at 10 µM and is consistent with previous work exploring cytosolic Ca^2+^ responses (35, 68). For qRT-PCR and Western blot analyses, ASM cells from nonasthmatics, asthmatics, and smokers were treated in the presence or absence of nicotine for 48 h.

### Transfection with siRNAs

ASM cells from the three patient groups were grown to ∼80% confluence at a density of 50,000 cells/well in a six-well plate and were treated with serum and antibiotic-free medium for 24 h before transfection. At the time of transfection, the medium in each well was replaced with 5 µM siRNA (DharmaFECT SMARTPool Cat. No. L-004143-00-0005) in 200 μL of reduced serum medium (DMEMF/12) and cells were transfected with DharmaFECT Transfection reagent (Cat. No. T-2001-02) according to the manufacturer’s protocol. Transfection with a scrambled (nontargeting RNA, Cat. No. D-001810-10-05) was used for controls. The volume in each well was made up to 2 mL with reduced serum medium and incubated for 24 h. After 24 h of transfection, the medium was removed, and the cells were replenished with 2 mL of serum-containing medium. The siRNA formulations were incubated with cells for additional 24 h under the same culture conditions before being examined to ensure the efficiency of knockdown by Western blot analysis.

### qRT-PCR Analysis

Cells were washed with RNA-grade DPBS, trypsinized, and centrifuged. Total RNA was extracted from cells using Quick-RNA MiniPrep kit (Zymo Research, Irvine, CA) following manufacturer’s protocol, and complementary DNA was synthesized using OneScript cDNA Synthesis kit (Richmond, BC, Canada). Standard qPCR techniques were followed (optimized for Roche LC480 Light Cycler) using QuantStudio 3 RT-PCR system as per the manufacturer’s instructions. The following primers were obtained from Integrated DNA Technologies (Coralville, IA) and used for qRT-PCR analysis as shown in Table 1. Fold changes in mRNA expression were calculated by normalizing the cycle threshold [C(t)] value of target genes to reference gene *S16* using the ΔΔCt method.

**Table 1.** Primers used for qRT-PCR

Gene	Primer Name	Sequence (5′–3′)
*Mfn1*	Mfn1-F	TCT CTCCGAGATAGCACCTCACCAAT
	Mfn1-R	TGCTGGCTAAGAAGGCGATTACTGCA
*Mfn2*	Mfn2-F	CCTGCTCTTCTCTCGATGCAACTCTA
	Mfn2-R	CTGCATTCCTGTACGTGTCTTCAAGG
*Opa1*	Opa1-F	TTTGCGGAGGACAGCTTGAGGGTTAT
	Opa1-R	CTCTTTTTCCAGTCTGGACCCACCAT
*Drp1*	Drp1-F	CACTTGTGGATTTGCCAGGAATGACC
	Drp1-R	TGCGACCATCTGGATCTACCTCTCTT
*Fis1*	Fis1-F	TCTGTGGAGGACCTGCTGAAGTTTGA
	Fis1-R	CAGGTAGAAGACGTAATCCCGCTGTT
*S16*	S16-F	CAATGGTCTCATCAAGGTGAACGG
	S16-R	CTGGATAGCATAAATCTGGGC

F, forward; R, reverse.

### Protein Analyses

hASM cell lysates were prepared using cell lysis buffer (Cell Signaling Technologies, Beverly, MA) containing protease and phosphatase inhibitors using previously described methods. Resultant supernatants were assayed for total protein content using the DC protein assay kit (BioRad, Hercules, CA). Approximately 30 µg of each lysate were loaded on 10% SDS-page and transferred onto 0.22 µm PVDF membranes. Nonspecific binding was blocked using 5.0% bovine serum albumin (BSA) and membranes probed overnight at 4°C with antibodies of interest. Blots were then incubated with Li-Cor secondary antibodies. Protein expression detection and densitometry were performed on a Li-Cor Odyssey IR scanning system (Lincoln, NE). Band intensities were normalized against β-actin.

Protein expression was also measured using the JESS, a capillary-based immunoblotting system (ProteinSimple, San Jose, CA). Following the manufacturer’s instructions, 0.3 µg cellular protein was loaded into 12-kDA to 230-kDa JESS separation modules with appropriate primary and secondary antibodies validated for JESS system. All antibodies targeting mitochondrial fusion and fission proteins were used at 1:50 dilution. Protein expression was normalized to total protein loading control. Digital representations of the electropherograms were used and then quantified using Compass for Simple Western Software. Internal consistency of protein expression measurement by traditional immunoblotting versus capillary-based approach was verified. Antibodies used for protein expression by Western blotting and JESS analyses are shown in Table 2.

**Table 2.** Antibodies for Immunoblotting (Western) and capillary electrophoresis (JESS)

Protein	Catalog Number	Dilution	Application	Manufacturer
Mfn1	ab129154	1:1,000 1:50	Western blotting JESS	Abcam
Mfn2	ab205236	1:1,000 1:50	Western blotting JESS	
Opa1	ab157457	1:1,000 1:50	Western blotting JESS	
Drp1	ab184247	1:1,000 1:50	Western blotting JESS	
Fis1	ab156865	1:1,000 1:50	Western blotting JESS	
α7nAChR	ab216485	1:100 1:50	Western blotting JESS	
β-Actin	G046	1:3,000	Western blotting	Abm Goods

### [Ca^2+^]_m_ Imaging

The techniques for real-time fluorescence [Ca^2+^]_m_ measurements in ASM have been previously described (69, 70). Briefly, hASM cells plated in eight-well chambered coverslips (Ibidi, Grafelfing, DEU) were incubated in Hanks’ balanced salt solution (HBSS) containing 1 μM Rhod 2-AM (Thermo Fisher Scientific, Waltham, MA) for 45 min at room temperature, medium aspirated, and washed three times in HBSS and incubated further for 15 min to allow for deesterification of the Rhod 2. Cells were then imaged at 1 Hz, 200-ms exposure using Nikon Eclipse Ti imaging system, LED fluorescence light source, and 16-bit high-sensitivity charge-coupled device (CCD) camera with excitation wavelength of 555 nm and emission detected at 580 nm using a 560/55 nm bandpass filter. Baseline fluorescence was established for 30–50 s followed by exposure to nicotine and [Ca^2+^]_m_ responses monitored in 10–15 cells/chamber using 20 individual, software-defined regions of interest. Inhibitors/activators were added before nicotine exposure (see RESULTS for details).

### Measurement of Mitochondrial Respiration

hASM cells were seeded on 24-well XF-24 plates (SeaHorse Biosciences, Billerica, MA) at 20,000/well density in DMEMF/12 media supplemented with FBS and AbAm in an XFp 24-well plate. After 24 h, cells were exposed to serum-free medium, before exposure to nicotine. Before measuring oxygen consumption rates (OCR; an indicator of mitochondrial respiration), the probe plate was hydrated with the XF Calibrant in a CO_2_-free incubator. The assay phenol red-free solution containing 10 mM glucose, 2 mM glutamine, 1 mM pyruvate, and 5 mM HEPES was kept in a 37°C CO_2_-free incubator to maintain the pH value. Next day, the XF Calibrant in the hydration plate was replaced with calibration solution and kept in a 37°C CO_2_-free incubator for 60–90 min. On the day of the assay, the cell culture medium was replaced with pH 7.4 phenol red-free assay solution and placed in a 37°C CO_2_-free incubator for 1 h. Another 24 h later, OCR was measured in all groups using the XFe24 extracellular flux analyzer (SeaHorse Biosciences). OCR measurements were acquired in the presence of 10 mM glucose, before (basal OCR) and after mitochondrial respiration inhibitors were injected into the system. The inhibitors used were: 1 µM Oligomycin-ATP synthase (Complex V) inhibitor (ATP uncoupler), 01.25 µM FCCP-ATP uncoupler (carbonyl cyanide p-trifluoromethoxyphenylhydrazone; drives maximum O_2_ consumption), and 1 µM antimycin A (a Complex III inhibitor) and 1 µM rotenone (a Complex I inhibitor), allowing for determination of respiration OCR’s, which were normalized for cell count using Hoechst staining on Cytation5 measured post hoc. All data were analyzed using Wave Software version 2.6.3.

### Mitochondrial Fission-Fusion Morphology

Human ASM cells were seeded in eight-well chambers at a density of 3,000 cells/well and grown as previously described. Following 48 h of treatment with nicotine (10 µM), the cells were loaded with 50 nM MitoTracker Green (Life Technologies Corporation, Eugene, OR; Cat. No. M7514) in HBSS for 30 min at 37°C in a non-CO_2_ incubator. Cells were then washed three times in HBSS and visualized using a Nikon Eclipse Ti imaging system with a 40×/1.30 NA Nikon oil immersion lens, an LED fluorescence light source with excitation/emission wavelengths of 488 nm/515 nm, respectively, and 16-bit high-sensitivity CCD camera. Single-cell images for mitochondrial morphology analysis were preprocessed with ImageJ software (NIH, Bethesda, MD) to correct for background fluorescence, brightness, and contrast. Images were then analyzed for form factor, an indicator of mitochondrial branching, and aspect ratio, an evaluation of mitochondrial branch length, using the MitoMorph toolset with a minimum of 60 cells analyzed/group.

### Statistical Analysis

All experiments were performed in hASM cells from at least five patients for each normal, asthmatic, and smoker groups and performed in duplicate for each patient sample for qRT-PCR and Western analyses. Statistical comparisons were made using either Student’s *t*-test, one-way ANOVA, or two-way ANOVA as appropriate, followed by Bonferroni post hoc multiple comparisons test using GraphPad Prism version 8.0.0 for Windows (GraphPad Software, San Diego, CA). Statistical significance was tested at a level of *P* < 0.05. All values are expressed as means ± SE. “*n*” values represent numbers of patients’ samples.

## RESULTS

### Nicotine Alters Mitochondrial Fusion Versus Fission

**Figure 1.**
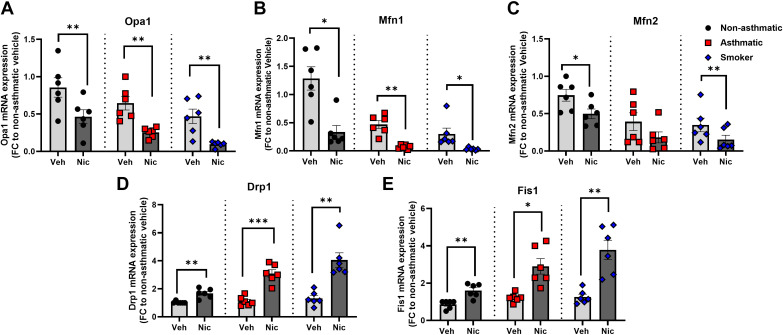
Nicotine and mitochondrial fusion vs. fission mRNA expression studies. qRT-PCR expression of mitochondrial fusion [Opa1 (*A*), Mfn1 (*B*), and Mfn2 (*C*)] and fission genes [Drp1 and Fis1 (*D* and *E*, respectively)] in nonasthmatic vs. asthmatic vs. smoker patients. Data are expressed as means ± SE. *P < 0.05, **P < 0.01, ***P < 0.001 vs. respective nonasthmatic/asthmatic/smoker vehicle; *n* = 6. Drp1, dynamin-related protein 1; Fis1, fission protein1; Mfn1, Mfn2, mitofusin 1 and 2; Opa1, optic atrophy 1.

To determine the mechanisms by which nicotine alters mitochondrial dynamics in hASM, we first examined expression of proteins known to regulate mitochondrial fission and fusion. In general, mitochondrial fusion is regulated by key mitofusins (Mfn1 and Mfn2) and the optic atrophy 1 protein (Opa1). We observed significant decreases in Mfn1 and Mfn2 gene expression (Fig. 1, *A*–*C*) for hASM from nonasthmatics (*P* < 0.05 for both), asthmatics (*P* < 0.01 for Mfn1), and smokers (*P* < 0.05 for Mfn1 and *P* < 0.01 for Mfn2). We also found significant reduction in Opa1 mRNA expression in samples treated with nicotine as compared with their respective vehicles for nonasthmatics, asthmatics, and smokers (*P* < 0.01). Such decreases in mitochondrial fusion parameters were significant for all samples treated with nicotine, albeit more so for the smokers and asthmatics. (Percent changes were 50%, 68%, and 16% for nonasthmatics as compared with 53%, 49%, and 44% for asthmatics and 66%, 39%, and 39% for smokers for fusion genes, *Opa1*, *Mfn1*, and *Mfn2*, respectively). Conversely, nicotine exposure upregulated mitochondrial fission genes as revealed by altered expression of dynamin-related *protein-1* (Drp1) and Fission 1 protein (Fis1) (Fig. 1, *D* and *E*). In hASM cells from nonasthmatics, 6 h of nicotine exposure induced significant increases in mRNA expression of Drp1 and Fis1 for nonasthmatics (*P* < 0.01 for both), asthmatics (*P* < 0.001 for Drp1 and *P* < 0.05 for Fis1), and smokers (*P* < 0.01 for both). With regard to fission genes, we observed increased percent changes in asthmatics and smokers exposed to nicotine. We found 50% and 47% change in Drp1 and Fis1 for nonasthmatics, 66% and 47% for asthmatics, and 62% and 75% for asthmatic Drp1 and Fis1 gene expression, respectively.

**Figure 2.**
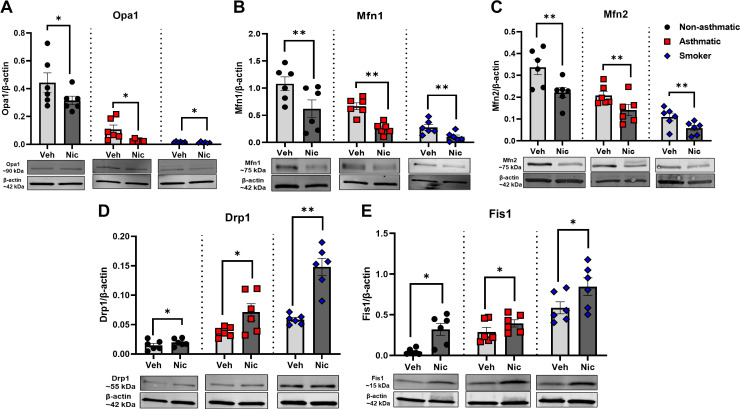
Nicotine and mitochondrial fusion vs. fission protein expression studies. Western blot expression of mitochondrial fusion [Opa1 (*A*), Mfn1 (*B*), and Mfn2 (*C*)] and fission proteins [Drp1 and Fis1 (*D* and *E*, respectively)] in nonasthmatic vs. asthmatic vs. smoker patients along with their representative Western blots are shown. Data are expressed as means ± SE. *P < 0.05, **P < 0.01 vs. respective nonasthmatic/asthmatic/smoker vehicle; *n* = 6. Drp1, dynamin-related protein 1; Fis1, fission protein1; Mfn1, Mfn2, mitofusin 1 and 2; Opa1, optic atrophy 1.

Protein expression studies (Fig. 2, *A* and *B*) showed trends similar to that of mRNA upon nicotine exposure, with downregulated expression of Opa1, Mfn1, and Mfn2 proteins in nonasthmatics (*P* < 0.05 for Opa1 and *P* < 0.01 for Mfn1 and Mfn2), asthmatics (*P* < 0.05 for Opa1 and *P* < 0.01 for Mfn1 and Mfn2), and smokers (*P* < 0.05 for Opa1 and *P* < 0.01 for Mfn1 and Mfn2). At the protein level, percent changes for nonasthmatics observed were 25%, 50%, and 28%, respectively, for Opa1, Mfn1, and Mfn2, whereas for asthmatics and smokers, there were significantly higher percent changes of 90%, 95%, and 25% and 95%, 75%, and 66%, respectively. Representative immunoblots for these groups are as shown. Similarly, we observed similar increase in protein expression of Drp1 and Fis1 (Fig. 2, *D* and *E*) in nonasthmatics (*P* < 0.05 for Drp1 and Fis1), asthmatics (*P* < 0.05 for Drp1 and Fis1), and smokers (*P* < 0.01 for Drp1 and *P* < 0.05 for Fis1). At the protein level, we found highest percent changes for nonasthmatic ASM exposed to nicotine wherein we found 66% and 97% change for Drp1 and Fis1 protein expression. On the other hand, there were subtle percent changes in asthmatics exposed to nicotine with 33% and 37% for Drp1 and Fis1 protein expression, respectively. Smoker ASM also showed significant percent changes with 66% for Drp1 and 44% for Fis1, respectively.

**Figure 3.**
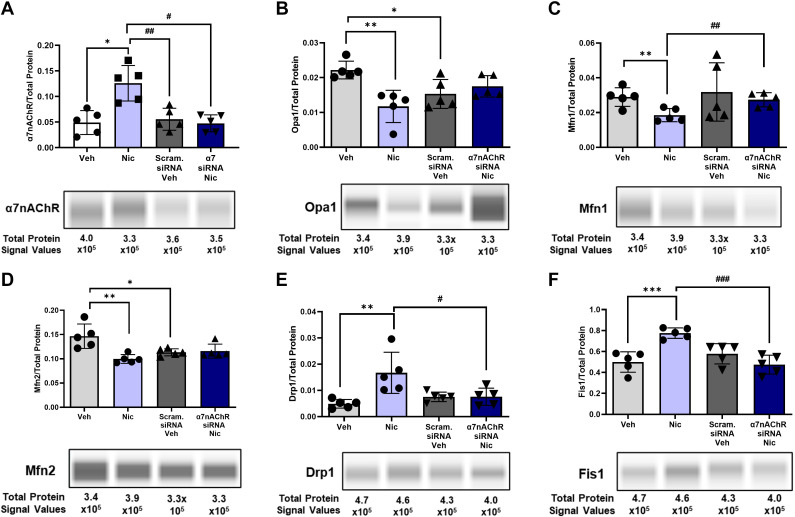
Nicotine-induced fusion-fission dynamics in the absence of α7nAChR. *A*: α7nAChR knockdown hASM was additionally verified using JESS immunoassay technique. Fusion proteins were tested for the expression of Opa1 (*B*), Mfn1 (*C*), and Mfn2 (*D*) in the presence of nicotine using scrambled (scram) and α7nAChR siRNA hASM cells. Fission proteins, Drp1 (*E*) and Fis1 (*F*), were also tested for their expression upon silencing α7nAChR. Data are expressed as means ± SE. *P < 0.05, **P < 0.01, ^***^P < 0.001 vs. respective nonasthmatic/asthmatic/smoker vehicle, #*P* < 0.05, ##*P* < 0.01, ###*P* < 0.001 vs. nicotine; *n* = 5–6. α7nAChR, alpha7 subtype nicotinic acetylcholine receptor; Drp1, dynamin-related protein 1; Fis1, fission protein1; hASM, human ASM; Mfn1, Mfn2, mitofusin 1 and 2; Opa1, optic atrophy 1.

Based on our previous findings of α7nAChR in hASM and its functional role in regulating [Ca^2+^]_i_ and ASM contraction as measured by traction force microscopy (35, 68), we explored the role of α7nAChR per se in nicotine effects on mitochondria using α7nAChR siRNA transfection (Supplemental Fig. S1; *P* < 0.001) of nonasthmatic hASM cells followed by chronic nicotine exposure. We also observed a significant downregulation of α7nAChR expression in α7nAChR siRNA-transfected hASM cells when exposed to nicotine, confirming the knockdown of α7nAChR due to attenuation of nicotinic activity (Fig. 3*A*; *P* < 0.05). Chronic nicotine exposure induced mitochondrial fragmentation as reflected by greater levels of fission proteins and reduction in fusion proteins (as shown in Fig. 2). We observed a reversal of these nicotine effects with α7nAChR siRNA, particularly for Mfn1 (Fig. 3*C*; *P* < 0.01), Drp1 (Fig. 3*E*; *P* < 0.05), and Fis1 (Fig. 3*F*; *P* < 0.001) using JESS detection technique—a capillary-based immunoassay system.

### Effects of Nicotine on Mitochondrial Morphology

**Figure 4.**
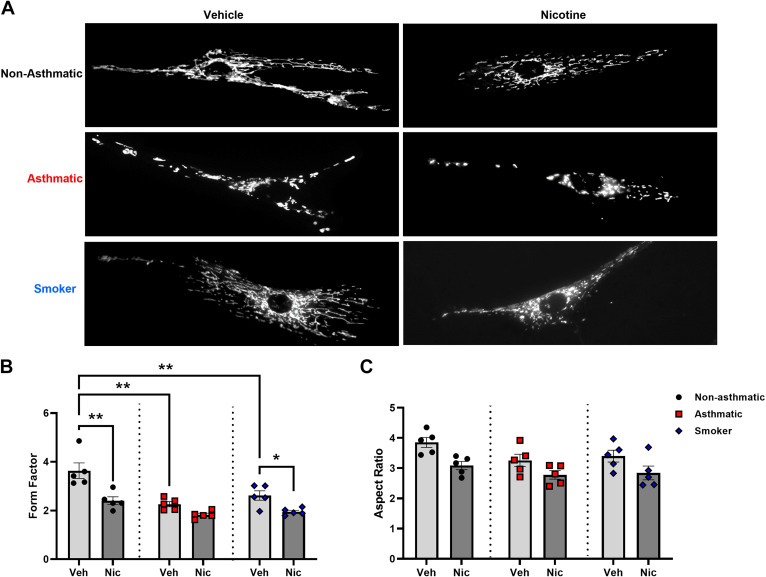
Nicotine effects on mitochondrial morphology. *A*: chronic nicotine exposure increased or more punctate mitochondrial appearance in human ASM cells from nonasthmatics, asthmatics, and smokers. *B*: exposure to nicotine further decreased form factor for nonasthmatics, asthmatics, and smoker ASM cells; this decreased form factor was evident at baseline in asthmatic and smoker human ASM cells compared with respective untreated nonasthmatic ASM. *C*: aspect ratio also appears to be reduced in nicotine exposed human ASM from nonasthmatics, asthmatics, and smokers, albeit not significantly. Data are expressed as means ± SE. *P < 0.05, **P < 0.01 vs. respective nonasthmatic/asthmatic/smoker vehicle; *n* = 5. ASM, airway smooth muscle.

ASM cells isolated from patients who were nonasthmatic, asthmatic, and current or recent smokers were exposed to the presence or absence of 10 µM nicotine for 48 h, stained with Mitotracker Green, and mitochondrial form factor and aspect ratio were analyzed as markers for mitochondrial morphology. Cells from untreated nonasthmatic controls typically demonstrated elongated mitochondrial tracks with complex, networked morphology, whereas cells from asthmatics and smokers displayed a significantly higher or increased punctate mitochondrial appearance (Fig. 4*A*). Exposure to 10 µM nicotine resulted in significant decrease in form factor (Fig. 4*B*; *P* < 0.01) and substantial reduction in aspect ratio, suggesting increased mitochondrial fragmentation when compared with untreated controls. In contrast, untreated ASM cells from both asthmatic patients and smoker patients with a recent history of smoking demonstrated a high level of mitochondrial fragmentation with significant (Fig. 4*B*; *P* < 0.01) decrease in form factor compared with untreated nonasthmatic patients. Nicotine exposure in asthmatics and smokers resulted in a further decrease in form factor (Fig. 4*B*; *P* < 0.01 in smokers) and aspect ratio compared with their respective untreated controls, with the mitochondria increasingly having a punctate appearance, indicating dismantling of the mitochondrial networks and branches. Likewise, the aspect ratio from both asthmatics and smokers was decreased compared with nonasthmatic controls, though not statistically significant (Fig. 4*C*).

### Nicotine and Mitochondrial Ca^2+^ in hASM

**Figure 5.**
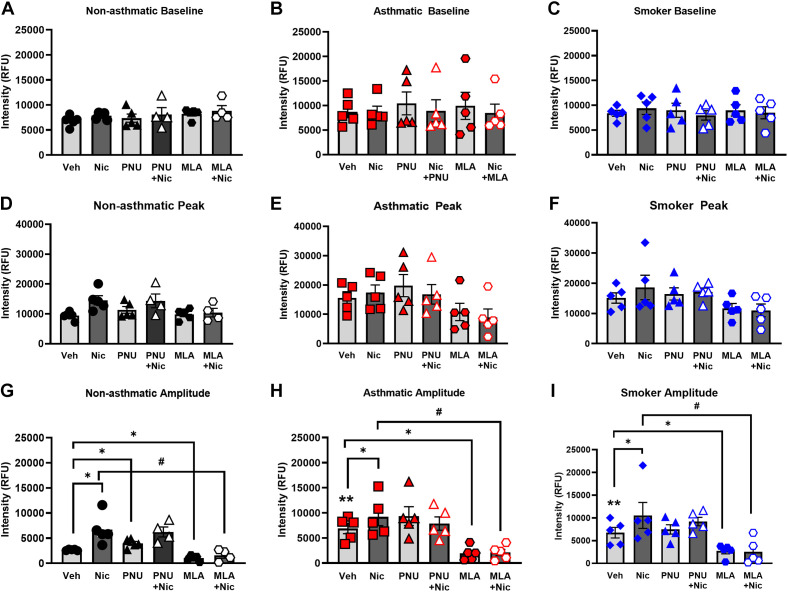
Effects of chronic nicotine exposure on ASM mitochondrial calcium in the presence of agonist and antagonist. Chronic nicotine exposure increased baseline [Ca^2+^]_m_ responses for nonasthmatic, asthmatic, and smoker hASM as shown in *A*, *B*, and *C*. The peak [Ca^2+^]_m_ responses tend to be higher for asthmatics and smokers as shown in *D*, *E*, and *F*. The [Ca^2+^]_m_ amplitude responses are significantly higher than normal for asthmatic and smoker hASM, further potentiated in the presence of agonist, PNU and nicotine and separately in the presence of the antagonist, MLA (*G*, *H*, and *I*). Data are expressed as means ± SE. *P < 0.05, **P < 0.01 vs. respective nonasthmatic/asthmatic/smoker vehicle, #*P* < 0.05 vs. nicotine; *n* = 5. ASM, airway smooth muscle; hASM, human ASM; MLA, methyllycaconitine.

We observed no significant difference in baseline [Ca^2+^]_m_ levels between the groups (Fig. 5, *A*–*C*). Although there was a trend toward higher peak [Ca^2+^]_m_ levels for asthmatics (but not smokers), these were not statistically significant (Fig. 5, *D–F*). However, significant increases in the amplitudes of [Ca^2+^]_m_ responses were noted for both asthmatics and smokers in comparison with nonasthmatics following acute nicotine exposure (Fig. 5, *G–I*; *P* < 0.01).

Preincubation with the α7nAChR potentiator PNU for 15 min significantly increased [Ca^2+^]_m_ responses to acute nicotine exposure in nonasthmatic hASM cells (Fig. 5*G*, P < 0.05). PNU-induced increases in [Ca^2+^]_m_ responses in hASM from both asthmatics and smokers were not noted to be significantly higher when compared with nonasthmatics (Fig. 5, *H* and *I*). In contrast, preexposure to the α7nAChR antagonist MLA in nonasthmatics, asthmatics, and smokers all significantly decreased [Ca^2+^]_m_ responses to subsequent acute nicotine exposure (Fig. 5, *G*–*I*; *P* < 0.05).

**Figure 6.**
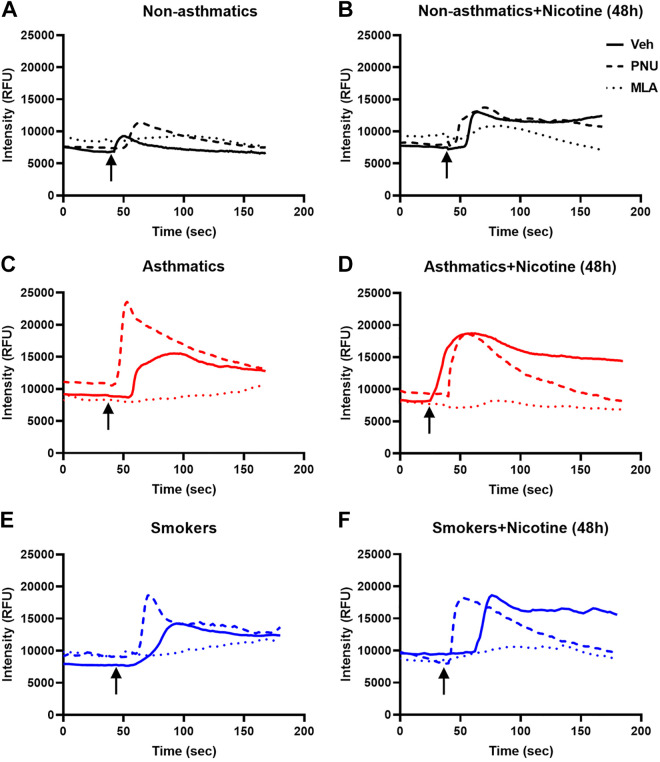
Effect of nicotine on mitochondrial calcium in human ASM. *A*, *C*, and *E*: exposure to acute nicotine produced increased [Ca^2+^]_m_ in ASM, an effect which is enhanced in the presence of α7nAChR agonist, PNU and inhibited in the presence of α7nAChR antagonist, MLA. Asthmatic and smoker ASM demonstrate an increase response to acute nicotine compared with nonasthmatic controls. *B*, *D*, and *F*: chronic nicotine enhances [Ca^2+^]_m_ in nonasthmatic and smoker ASM, effects again inhibited by MLA. Tracings are representative of responses close to the average. α7nAChR, α7 subtype nicotinic acetylcholine receptor; ASM, airway smooth muscle; MLA, methyllycaconitine.

Chronic nicotine exposure in nonasthmatic hASM demonstrates significantly increased subsequent [Ca^2+^]_m_ responses to acute nicotine when compared with untreated controls (Fig. 5*G*; *P* < 0.05). A similar potentiation of [Ca^2+^]_m_ responses to acute nicotine was observed for hASM from asthmatics and smokers preexposed to chronic nicotine (Fig. 5, *H* and *I*; *P* < 0.05), although such potentiation was comparable across nonasthmatics, asthmatics, and smokers. Although PNU did not further potentiate chronic nicotine effects (compared with PNU in vehicle-exposed cells), the α7nAChR inhibitor MLA did suppress such effects, albeit to comparable levels between nonasthmatics, asthmatics, and nonsmokers (Fig. 5, *G*–*I*; *P* < 0.05). Exposure to chronic nicotine increased [Ca^2+^]_m_ at baseline for nonasthmatic, asthmatic, and smoker ASM (Fig. 6, *A*, *C*, and *E*). This effect was observed to be higher at baseline for asthmatic and smoker ASM compared with nonasthmatics. Preexposure to α7nAChR agonist, PNU, potentiated [Ca^2+^]_m_ as observed for all nonasthmatic, asthmatic, and smoker ASM at baseline, and almost reaching its peak when compared with chronic nicotine exposure (Fig. 6, *B*, *D*, and *F*). Conversely, α7nAChR inhibitor, MLA, diminished [Ca^2+^]_m_ sensitivity for subsequent reduced [Ca^2+^]_m_ influx as observed across nonasthmatic, asthmatic, and smoker.

**Figure 7.**
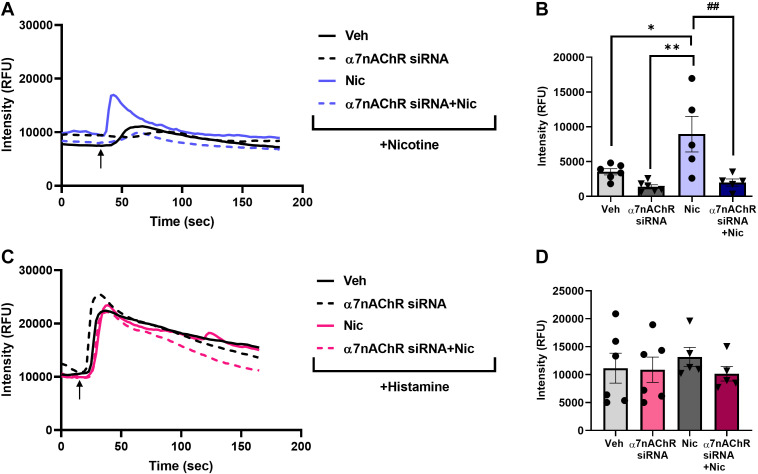
Effects of nicotine on [Ca^2+^]_m_ in the absence of α7nAChR. Effect of chronic nicotine exposure on [Ca^2+^]_m_ using α7nAChR siRNA was determined both in the presence of nicotine and histamine on hASM. Representative tracings of nicotine in the presence of α7nAChR siRNA showed no increases in Ca^2+^]_m_ (*A* and *B*), whereas histamine had no effect on α7nAChR siRNA in hASM (*C* and *D*). Data are expressed as means ± SE. *P < 0.05, **P < 0.01 vs. respective nonasthmatic/asthmatic/smoker vehicle, ##*P* < 0.01 vs. nicotine; *n* = 5–6. α7nAChR, alpha7 subtype nicotinic acetylcholine receptor; hASM, human ASM.

As with the mitochondrial fission/fusion parameters, we tested the role or α7nAChR in nicotine effects on [Ca^2+^]_m_ using α7nAChR siRNA. The effects of chronic nicotine exposure alone as well as acute nicotine (regardless of chronic exposure) were significantly blunted by α7nAChR siRNA (Fig. 7, *A* and *B*; *P* < 0.01). To ensure specificity of such effects for nicotine, we tested an agonist such as histamine (10 μM) that acts entirely independent of α7nAChR and found that the siRNA did not appreciably impact on [Ca^2+^]_m_ responses (Fig. 7, *C* and *D*).

### Nicotine Disrupts hASM Mitochondrial Bioenergetics

**Figure 8.**
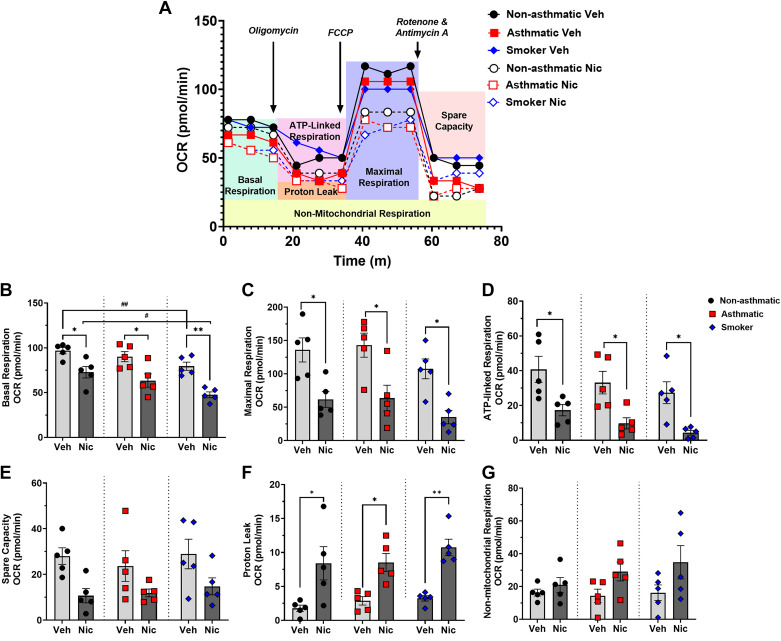
Nicotine effects on mitochondrial respiration parameters. Energy metabolism in nonasthmatic/asthmatic/smoker hASM vs. chronically exposed nicotine hASM was impaired as shown in *A*. Mitochondrial oxygen consumption rate (OCR) was measured using a Seahorse XFp analyzer for the following parameters: basal respiration (*B*), maximal respiration (*C*), ATP-linked respiration (*D*), spare capacity (*E*), proton leak (*F*), and nonmitochondrial respiration (*G*). Data are expressed as means ± SE. *P < 0.05, **P < 0.01 vs. respective normal/asthmatic/smoker vehicle, #*P* < 0.05, ##*P* < 0.01 vs. counterpart treatment group; *n* = 5. hASM, human ASM.

Seahorse analysis (Fig. 8*A*) of OCR showed that hASM cells from asthmatics and particularly smokers had significantly lower basal respiration (Fig. 8*B*; *P* < 0.01 for smokers) and maximal respiration (Fig. 8*C*). Chronic exposure to 10 µM nicotine further substantially blunted both parameters in all three groups (Fig. 8, *B* and *C*; *P* < 0.05 and *P* < 0.01 for smokers’ basal respiration alone), and additionally reduced ATP-linked respiration (Fig. 8*D*; *P* < 0.05) and spare capacity (Fig. 8*E*) again in the three groups. Effects on the latter two parameters with nicotine were particularly noted for the smoker group. We also observed a trend toward increase in proton leak, significant especially for hASM from smokers (Fig. 8*F*; *P* < 0.05 for nonasthmatics and asthmatics and *P* < 0.01 for smokers) and an increasing trend for nonmitochondrial (Fig. 7*G*) respiration, albeit not significantly so for nicotine-exposed hASM across the three groups.

## DISCUSSION

Given known effects of smoking and secondhand smoke exposure (71–73) and now even e-cigarettes that contain nicotine (9–14, 19) on asthma, the mechanisms by which nicotine acts on airway cells become significant toward alleviating detrimental effects and development of novel therapeutics. Although it is generally believed that ACh exerts bronchoconstrictive effects via muscarinic receptors on ASM, and that nAChRs are more typical of preganglionic neuronal function (25–27, 36), our previous data in hASM showed the presence of functional nAChRs (35), particularly α7nAChR, with effects on [Ca^2+^]_i_ (34). We had also previously shown that cigarette smoke and asthma-relevant cytokines increase α7nAChR expression (35) and that activation of ASM nAChRs increases [Ca^2+^]_i_ and cellular contractility to a greater extent in asthmatic ASM (34), highlighting the importance of hASM nAChRs and of nicotinic receptor activation in asthma pathophysiology. Our present study demonstrates that beyond acute effects on [Ca^2+^]_i_ and contractility, nicotine, particularly with chronic exposure, impacts mitochondrial structure and function, resulting in increased mitochondrial fission and disrupted oxidative phosphorylation. Furthermore, these effects are more prominent in ASM from asthmatics and interestingly from smokers, suggesting long-term nicotine effects on mitochondria. The relevance of these results lies in the potential for disruption of mitochondrial structure and functionality to permit cytosolic Ca^2+^ to remain elevated with nicotine exposure, leading to long-term downstream genomic and nongenomic changes in ASM that overall contribute to airway hyperreactivity and remodeling. Thus, targeting mitochondria may represent a novel approach to alleviating nicotine effects in the airway.

There has been substantial work describing the 16 nAChR subunits and how they create different pentameric configurations resulting in different permeabilities and channel properties (24, 35–37, 74). Of most relevance, we previously found that the “neuronal” subunit α7 that forms homopentameric nAChRs is highly expressed in hASM (35, 68). The relevance of α7nAChRs per se for ASM lies in the high Ca^2+^ permeability, as also demonstrated by the modulation of [Ca^2+^]_i_ in hASM (34) and thus a potentially procontractile effect toward airway reactivity. Indeed, in previous studies, we noted increased [Ca^2+^]_i_ as well as ASM cellular contractility as determined by traction force microscopy following nicotine exposure that is dependent on α7nAChR (35, 68). Certainly, ASM is not the only airway cell type expressing α7nAChR, which is also present in epithelium and macrophages (39, 40, 75, 76). In epithelial cells, α4, α5, or α7 subunit expression correlates with lung function (39, 76). Therefore, these findings may also be particularly relevant in other cell types residing within the airways. Furthermore, nicotine itself can enhance α7 subunit expression. Although it is likely that different cell types express a range of nAChR subunits, we previously showed that α4nAChR expression is much lower in hASM compared with α7nAChR (35). Importantly using coimmunoprecipitation, we had found that α4 and α7 could interact with β2 subunits (35). These initial findings are entirely consistent with the functional relevance of α7- and β2-mediated effects of nicotine in hASM, particularly in asthmatics, and justify the focus on α7nAChR in the current study.

Mitochondrial dysfunction is garnering increasing interest in the pathophysiology of asthma (50, 58, 59). With mitochondria being the major source of reactive oxygen species (ROS), and ROS being a downstream effect of inflammation, mitochondrial dysfunction becomes relevant toward understanding asthma pathophysiology. Mitochondrial dysfunction involves complex biochemical, structural, and metabolic features (59, 77, 78) that are often interlinked. Thus, the present study aims to preliminarily understand the impact of nicotine on these features toward a larger appreciation of how chronic nicotine exposure in the context of smoking or e-cigarette exposure could impact on ASM structure and function.

It is now recognized that mitochondrial structure is dynamically regulated and disrupted in disease (49) with multiple mechanisms that control mitochondrial morphology and networking (fusion), disruption and fragmentation (fission), and biogenesis and motility within the cell. Regulation of mitochondrial networks is critical for their interactions with the plasma membrane, endoplasmic reticulum, and other structures not only to optimize energy production and availability but also to allow mitochondria to influence other cellular functions such as Ca^2+^ regulation, synthetic function, and proliferation for example. Mitochondrial fusion and fission proteins or machineries, therefore, do not function as independent units, but are rather integrated with bioenergetics, signaling, and other functions within the cell (79). Thus, maintenance of mitochondria structural integrity is essential for cell survival and normal growth. Here, mitochondrial fission versus fusion play key roles where an imbalance in fusion-fission leads to impaired [Ca^2+^]_m_, increased ROS generation, and promotion of apoptosis. During an active or healthy respiratory state, mitochondrial fusion is preferred by the cell, whereas under states of stress, injury, or hyperinflammation, the cell works toward removing the damaged mitochondria (80). The mitofusins Mfn1 and Mfn2 on the OMM and Opa1 on the IMM are the two main classes of mitochondrial fusion proteins. A reduction in the expression and function of these fusion proteins results in mitochondrial fragmentation. This is previously shown by other groups in different cell types, including in hASM where factors such as cigarette smoke extract (CSE) or TNFα decrease expression of Mfn1, Mfn2, and Opa1 with increased fragmentation observed with mitochondrial imaging (70, 81). Similarly, mitochondrial fission proteins Fis1, which helps form focal rings around the mitochondria and recruits cytosolic Drp1 to the mitochondrial surface, enable mitochondrial fragmentation. It has been previously observed that CSE (81) and TNFα (70) can enhance Drp1 expression, consistent with increased mitochondrial fragmentation. Dysregulated mitochondria have been reported previously in epithelium and ASM of patients with asthma and in mouse models of allergic asthma (82–84). Changes in mitochondrial fusion and fission protein expression can have downstream cytosolic and nuclear signaling effects (78) and thus are of functional importance. Accordingly, our findings showing that nicotine, via α7nAChR, promotes mitochondrial fission, increasing fission regulators whereas decreasing fusion mechanisms, are significant toward understanding the chronic effects of nicotine that are mediated by mitochondrial structure. Here, an interesting observation is that hASM from patients with asthma and particularly from smokers show baseline lower levels of fusion proteins, but higher levels of fission proteins. In smokers at least, it is possible that such effects are a result of chronic nicotine exposure (or at least cigarette smoke), consistent with our previous results with CSE, and since in this study, we also observe that nicotine induces similar changes in hASM from nonasthmatics and exacerbates the differences in smokers. In patients with asthma, unless there is nicotine exposure in the patients from which samples were derived, it is difficult to determine the cause for baseline differences in mitochondrial fission/fusion regulators. However, chronic inflammation is a likely mechanism, evidenced also by previous data showing that cytokines such as TNFα (70) can induce changes in the fission/fusion balance.

Separately, other studies have explored the impact of cigarette smoke exposure on lung eosinophilia and reported an inflammatory-suppressive role and a reduced response to allergens (85). Interestingly, they also show that this effect is independent of α7nAChR, suggesting that other coincident interactions, such as with other resident cell types (including immune cells), may impact cell-specific effects of nicotine via α7nAChR function to determine final outcomes (85).

The current study did not explore upstream mechanisms by which nicotine modulates mitochondrial fission and fusion proteins. However, high [Ca^2+^]_i_ itself can be a factor inducing mitochondrial fragmentation, being normally suppressed at baseline Ca^2+^ levels (49). Such Ca^2+^-mediated effects may serve to optimize mitochondrial localization for energy utilization or other functions or may be an outcome of other changes induced by Ca^2+^. We recently showed that nicotine by itself can increase hASM [Ca^2+^]_i_, and can potentiate agonist-induced [Ca^2+^]_i_ responses (34), with such effects being more pronounced in asthmatic hASM. Accordingly, Ca^2+^ may be at least one mechanism by which nicotine, acting via α7nAChR, induces mitochondrial fission. Overall, these studies show that nicotine influences mitochondrial structure although we recognize it does not determine whether effects on structure are upstream to changes in mitochondrial function or are a consequence. It is possible that in the short term, changes in mitochondrial calcium or its disruption allows cytosolic calcium to remain elevated, leading to both genomic and nongenomic changes such as in the cytoskeleton and in production of mitochondrial structural proteins, leading to subsequent fission. In the long term, such fission and resultant disruption of mitochondrial calcium buffering may perpetuate the effect on fission.

We also did not explore downstream impact of nicotine-enhanced mitochondrial fission, particularly in asthmatics or smokers. The relevance of fission/fusion is clear during the cell cycle where mitochondria fragment and rejoin constitutively during the G0 phase (quiescent state) and fusion is increased in the interphase: processes that are reversed during the M phase when mitochondria get fragmented, mixed, and distributed equally among daughter cells. Thus, fission/fusion processes can influence cell proliferation, especially under the influence of stressors such as inflammation, ROS, or environmental factors. Nicotine has been shown to increase ASM proliferation (86, 87), and such effects may be a result of altered mitochondrial fission/fusion. Furthermore, as suggested earlier, fission may allow for disruption of mitochondrial calcium buffering, leading to a maintenance of higher cytosolic calcium or of processes such as ROS and enhanced proliferation. Interestingly, mitochondrial morphology changes are also accompanied by alterations in metabolic activity and respiration indicating an important, yet complex role that exposure to nicotine plays (88). Moreover, modulation of mitochondrial network morphology and mitochondrial biogenesis plays an important role in cellular responses to mitochondrial stress (89). Fasting or nutrient starvation is reported to induce mitochondrial elongation resulting in an ATP-driven cell death (90). However, changes in mitochondrial structure may be secondary (rather than causing) alterations in cell function and performance. Perhaps rather than energetic status, structural changes in mitochondria are more representative of transient cellular functions such as Ca^2+^ and ROS. However, given that nicotine may activate several cytosolic and mitochondrial (probably nuclear) pathways, perhaps with overlapping timelines, it is difficult to construe the “driver” of these changes. Nonetheless, since calcium changes are transient (seconds-minutes) versus fission/fusion and other bioenergetic changes, we speculate that [Ca^2+^]_m_ changes may be the key mechanism driving other processes.

We did observe increased [Ca^2+^]_m_ with nicotine exposure, particularly in asthmatics and smokers, which is characteristic of increased stress and inflammation. [Ca^2+^]_m_ is known to regulate respiration and other cellular buffering functions (91, 92). Indeed, mitochondrial structural proteins such as Mfn2 are thought to be important in mitochondria-ER interactions and in regulating mitochondrial Ca^2+^ buffering via the Ca^2+^ uniporter (62–64)_._ Whether reduced Mfn2 as observed in this study would lead to impaired mitochondrial Ca^2+^ buffering or enhanced Ca^2+^ uptake is not clear. Regardless, based on previous findings of increased cytosolic Ca^2+^ by nicotine, it is possible to hypothesize that such increases in [Ca^2+^]_i_ trigger mitochondrial Ca^2+^ uptake, especially if with chronic nicotine exposure, mitochondrial localization toward the plasma membrane or the ER would allow for rapid uptake of Ca^2+^ influx or intracellular release, respectively. Furthermore, increased [Ca^2+^]_m_ enhances mitochondrial fission and is associated with changes in homeostasis of fusion versus fission proteins (50, 65), and thus it may be that the altered fission with nicotine is downstream of nicotine effects on [Ca^2+^]_i_ and [Ca^2+^]_m_: aspects that remain to be explored.

One impact of indirect elevation of [Ca^2+^]_i_ (as a result of impaired [Ca^2+^]_m_) may be on mitochondrial structure itself. Studies in nonlung cells have investigated the effects of [Ca^2+^]_i_ on mitochondrial dynamics and respiration (93). [Ca^2+^]_i_ regulation has been shown to regulate mitochondrial fission in myocytes indicating that mitochondria can undergo rapid fragmentation in presence of elevated [Ca^2+^]_i_ (94). Similarly, an increase in [Ca^2+^]_i_ also occurs in states of high metabolic demand such as in cases where there is exaggerated mitochondrial fission and an imbalance in fusion versus fission processes (95, 96). This can also translate to an impaired buffering capacity of the mitochondria (as witnessed in insufficient basal respiration upon exposure to nicotine in our present study) due to reduced activity of the electron transport chain to power adequate local ATP across the plasma membrane (97). Thus, the interlinks between mitochondrial processes may further link to cytosolic calcium and its many other effects (97).

Regardless of the many roles that mitochondria are now thought to play in cell structure and function, mitochondrial energetics are obviously critical, and mitochondria rapidly increase energy production under conditions of stress (spare or bioenergetic respiratory capacity), critical for long-term cell survival and homeostasis. Mitochondrial fission/fusion proteins and [Ca^2+^]_m_ can each regulate energetics, but the links are not straightforward and were not explored in this study. Nonetheless, our studies showed substantial baseline differences in basal respiration, maximal respiration, and ATP-linked respiration between nonasthmatics versus asthmatics or particularly smokers. We further found nicotine effects in blunting of these parameters. Basal respiration is characteristic of the resting energetic measure of mitochondria (98, 99). We observed a lower basal respiration in nicotine-exposed hASM suggesting decreased pyruvate conversion from glycolysis. Accordingly, ATP production or ATP-linked respiration also decreased in nicotine-exposed hASM, albeit more so for smokers and asthmatic hASM. Addition of FCCP to induce maximal respiration also showed a blunting of the cellular response with nicotine. Reduced maximal respiration rate, like basal respiration rates, indicates that chronic nicotine has a strong inhibitory effect on the mitochondrial respiratory chain. Similarly, a reduction in ATP-linked respiration rate following nicotine suggests impaired utilization of glucose as the primary source of energy uptake and production by mitochondria (100, 101). Spare respiratory capacity that should be available under conditions of stress toward reestablishment of homeostasis (102) was also reduced by nicotine exposure, suggesting that mitochondria may be geared toward utilizing other nonconventional energy sources. Likewise, energy metabolism from unconventional sources leads to mitochondrial dysfunction indicated by an increased proton leak (deficient ATP production) and nonmitochondrial respiration (ATP generation by glycolysis) as we observed. Such nonmitochondrial respiration could be attributed to enzymes associated with inflammation such as cyclo-oxygenases, lipoxygenases, or NADPH oxidases that typically increase in response to stressors such as ROS. Furthermore, these results at a cellular level in ASM are akin to the model of a high-fat, low-carbohydrate diet in smokers on nicotine replacement therapy (103) where chronic nicotine results in decreased glycolysis thereby preventing use of glucose as an energy source, which synergistically with increased breakdown of fatty acids contributes to malnutrition of chronic smokers. Whether a similar model holds true for exacerbated asthma or airway dysfunction by smoking remains to be determined.

In summary, we present novel data on the presence of functional α7nAChRs in human ASM that contribute to altered mitochondrial structure and function reflected by [Ca^2+^]_m_ and respiratory responses, exacerbated in asthmatics and smokers. The significance of such findings lies in the potential for nicotine to directly affect the airway in the context of smoking or vaping, promoting AHR, and remodeling. Thus, understanding the importance of mitochondria is crucial to potentially developing mitochondrial-targeted therapy in alleviating the detrimental effects of nicotine.

## DATA AVAILABILITY

Data will be made available upon reasonable request.

## SUPPLEMENTAL DATA

10.6084/m9.figshare.22825145 Supplemental Fig. S1: https://doi.org/10.6084/m9.figshare.22825145.

## GRANTS

This work was supported by NIH Grants R01-HL142061 (to C.M.P. and Y.S.P.), R01-HL088029 (to Y.S.P.), and R01-HL146705 (to V.S.).

## DISCLOSURES

No conflicts of interest, financial or otherwise, are declared by the authors.

## AUTHOR CONTRIBUTIONS

N.A.B., M.A.T., C.M.B., V.S., Y.S.P., and C.M.P. conceived and designed research; N.A.B., M.A.T., and C.M.B. performed experiments; N.A.B., M.A.T., and C.M.B. analyzed data; N.A.B., M.A.T., and C.M.B. interpreted results of experiments; N.A.B., M.A.T., and C.M.B. prepared figures; N.A.B., M.A.T., C.M.B., V.S., Y.S.P., and C.M.P. drafted manuscript; N.A.B., M.A.T., C.M.B., V.S., Y.S.P., and C.M.P. edited and revised manuscript; N.A.B., M.A.T., C.M.B., V.S., Y.S.P., and C.M.P. approved final version of manuscript.
